# 2169. Anti-anaerobic Antibiotic Use is Associated With Lower Rehospitalization Rate in Known and Suspected Pleural Empyema in Adults

**DOI:** 10.1093/ofid/ofac492.1789

**Published:** 2022-12-15

**Authors:** Benjamin S Avner, Zeena Qiryaqoz, Justin Mak, James Le, Anush Ginosyan, Cuyler Huffman

**Affiliations:** Western Michigan University Homer Stryker MD School of Medicine, Kalamazoo, Michigan; Western Michigan University Homer Stryker MD School of Medicine, Kalamazoo, Michigan; Western Michigan University Homer Stryker MD School of Medicine, Kalamazoo, Michigan; Western Michigan University Homer Stryker MD School of Medicine, Kalamazoo, Michigan; Western Michigan University Homer Stryker MD School of Medicine, Kalamazoo, Michigan; Western Michigan University Homer Stryker MD School of Medicine, Kalamazoo, Michigan

## Abstract

**Background:**

There is not a prevailing consensus on appropriate antibiotic choice, route, and duration in the treatment of bacterial pleural empyema after appropriate source control. Professional society guidelines identify a wide range of antibiotic durations, and generally recommend an antibiotic with broad-spectrum activity against anaerobes (e.g. clindamycin, metronidazole, carbapenem, or penicillin/beta-lactamase inhibitor combination), while noting the lack of comparative trials with which to guide recommendations.

**Methods:**

We performed a hypothesis-generating retrospective chart review analysis of clinical outcomes in 355 adult inpatients who had pleural drainage, via either chest tube or surgical intervention, for known or suspected empyema. The aims of the investigation were to compare clinical outcomes with regard to 1) the use of antibiotics with broad activity against anaerobes, 2) total antibiotic duration, and 3) IV antibiotic duration. Mann-Whitney U test with Bonferroni multiplicity adjustment was used to compare outcomes with regard to these variables.

**Results:**

A longer course of an anti-anaerobic antibiotic was associated with both lower 30-day readmission rates for empyema (11.9 ± 13.8 antibiotic days in non-readmission group, n = 321 vs 2.2 ± 2.7 days in readmission group, n = 17, *p*-value 0.003) and lower all-cause 30-day readmission (12.3 ± 14.1 in non-readmission group, n = 290 vs 6.1 ± 7.9 in readmission group, n = 49, *p*-value 0.003). Longer total antibiotic duration was associated with lower empyema-specific 30-day readmission rates (21.5 ± 20.1 vs 12.4 ± 8.2, *p*-value 0.010), with a non-significant trend towards lower all-cause 30-day readmission (22.1 ± 20.9 vs 15.0 ± 8.3). IV antibiotic duration was not associated with a difference in 30-day readmission (data not shown).

**Conclusion:**

Our data support the premise that routine use of anti-anaerobic antibiotics is indicated in the treatment of pleural empyema. However, our study casts doubt on the benefits of extended IV rather than oral antibiotics in the treatment of empyema. This represents a target for future investigation with the aim of limiting complications associated with the excessive use of IV antibiotics.

**Disclosures:**

**All Authors**: No reported disclosures.

